# Are we underestimating exposures from NORM dust?

**DOI:** 10.1093/annweh/wxaf043

**Published:** 2025-08-05

**Authors:** Gregory Stanley Hewson, Martin Ian Ralph, Marcus Cattani

**Affiliations:** School of Medical and Health Sciences, Edith Cowan University, 270 Joondalup Drive, Joondalup, Western Australia 6027, Australia; School of Medical and Health Sciences, Edith Cowan University, 270 Joondalup Drive, Joondalup, Western Australia 6027, Australia; School of Medical and Health Sciences, Edith Cowan University, 270 Joondalup Drive, Joondalup, Western Australia 6027, Australia

**Keywords:** airborne radioactivity, dust exposure, mining, particle size distribution, personal air sampling

## Abstract

The inhalation of dust containing naturally occurring radioactive materials (NORM) associated with mining and mineral processing operations may lead to potential long-term health impacts, including cancer and chronic lung disease, due to alpha particle-emitting radionuclides. This study evaluates the effectiveness of air sampling strategies used to estimate radiation doses from NORM exposure, with a focus on the Western Australian minerals industry. The objectives were to review current sampling and analysis protocols, identify factors contributing to over- or underestimation of dose, and propose adjustments to improve intake assessments. A review of research and guidelines applicable to NORM dust exposure was conducted, and the sampling efficiency of the government-recommended 7-hole and IOM sample heads was compared, considering measured dust particle size distributions. Key inhalation-related parameters, including use of similar exposure group (SEG) mean concentrations, worker breathing rates, median dust particle size, and intake-to-dose conversion factors, were analysed to assess their influence on intake calculations. The findings indicate that use of the 7-hole sampler, currently recommended by local guidelines, may underestimate airborne radioactivity concentrations by 2-fold or more, primarily due to reduced sampling efficiency for larger particles. Standard default assumptions for breathing rates and aerosol characteristics used to convert the measured concentrations to intake and dose may further contribute to underestimation. This study recommends updating air sampling methods and dose assessment protocols to better align with workplace-specific exposure conditions and improve worker health protection in NORM industries.

**What’s important about this paper**

This study has identified that current practices in Western Australia for internal radiation dose estimates from intake of mineral dusts containing naturally occurring radioactive materials likely underestimate dose by 2-fold or greater. Recommendations are made to change sampling and analysis methods to ensure dose assessment protocols reflect realistic intake conditions, thereby improving exposure and risk assessments.

## Introduction

There are many industries where workers may be exposed to intake of naturally occurring radioactive materials (NORM), including mineral sands (ilmenite, rutile, zircon, and monazite) and rare earth processing, titanium dioxide and zirconia production, phosphate processing, oil and gas recovery processes, and production of metals such as tin, copper, niobium, and tantalum (ICRP 2019). In Western Australia, workers involved in operating mineral sands plants are exposed to a potential health risk arising from inhalation of alpha particle-emitting radionuclides associated with dust. Historical estimates from industry personal air sampling (PAS) programmes indicate that daily thorium (232Th) intake by workers in the 1980s and 1990s averaged about 0.5 Bq d^−1^ (120 µg d^−1^) (Hewson and Fardy 1993; Terry et al. 1997), while more recent estimates suggest a reduction to 0.1 Bq d^−1^ (24 µg d^−1^) (Ralph et al. 2024). However, concern has been expressed that these intake figures may underestimate the true intake levels (Hewson et al. 2025a). A review of technologically enhanced NORM (TENORM) highlighted the significant variability of doses calculated for TENORM-exposed workers and the need to improve the accuracy of exposure measurements (Vearrier et al. 2009).

Reliable airborne radioactivity data will be relevant to any future epidemiology, since past and current long-term (>10 yr) workers may suffer health consequences from thorium ore dust intakes that occurred decades earlier. Improving estimates of intake and radiation dose is achieved by ensuring the air sampling device is efficient in collecting the aerosol of interest, and site-specific intake parameters are used in dose assessment protocols. Routine exposure assessment in Western Australian NORM operations relies on PAS, which is broadly consistent with international practices (IAEA 2018). Prior to 1986, dust sampling for airborne radioactivity in the Western Australian minerals industry was done in different ways (ie using various dust collection devices) by industry practitioners, and this complicated the retrospective assessment of intake (Hewson and Terry 1995). Elimination of the earlier ad hoc sampling regimes used by the industry was achieved by standardisation of sampling practices by regulatory agencies (Hewson 1990). In particular, the referencing of aerosol conventions defined by the International Organisation for Standardisation, commencing in 1983 and updated periodically since then (ISO 1995).

Since 1986, the protocols for sampling and analysis of airborne radioactive dust in the Western Australian industry have remained essentially unchanged. Workers assigned to a similar exposure group (SEG) are randomly selected to wear a sampling train consisting of a small portable air sampling pump connected via a tube to a 7-hole sample head on occasional shifts over a reporting period (usually 1 yr). The collected dust sample is subsequently analysed for airborne radioactivity, typically total alpha activity concentration, measured in becquerels per cubic metre (Bq m^−3^).

The dose–response relationship for internal alpha emitters has not been derived from workplace measurements and instead is determined via the use of biokinetic and dosimetric models applied to the intake (ICRP 2015). In the case of inhalation exposure, the intake is taken to be the amount of radioactivity inhaled. Hence, it is important that the PAS technique collects the appropriate aerosol fraction. Converting the intake to dose requires knowledge of, amongst other things, the relative activities of the radionuclides inhaled, aerosol (dust) particle size, and solubility. Typically, default parameter values are used in the absence of site- or material-specific data, and the values selected may not be representative of actual exposure conditions, leading to over- or underestimation of dose. Additionally, assumptions regarding breathing rates, typically set at 1.2 m^3^ h^−1^ for a worker, may not accurately reflect the physical demands of mining work, especially during manual tasks.

This study aims to (1) evaluate the adequacy of current air sampling protocols for assessing NORM dust exposure, (2) identify factors contributing to dose under- or overestimation, and (3) propose practical adjustments to improve the accuracy of dose assessments, with broader implications for other NORM industries.

## Methodology

The study was conducted in 3 phases: (1) a review of publications from international and government agencies to identify recommended sampling strategies and appropriate methods and assumptions for converting NORM dust intake to dose, (2) a review of literature related to assessment of occupational NORM dust exposure, including past research in Western Australian mining operations and elsewhere relating to review of dust sampler performance, and (3) an analysis of key parameters influencing the calculation of internal radiation dose from airborne radioactivity concentration measurements.

### Literature reviews

The initial review focussed on identifying local (Western Australian government) guidelines, Australian and international standards, and publications from international radiation agencies such as the International Commission on Radiological Protection (ICRP) and the International Atomic Energy Agency (IAEA) applicable to internal dose assessment following intake of NORM dust/aerosols.

**Figure 1.**
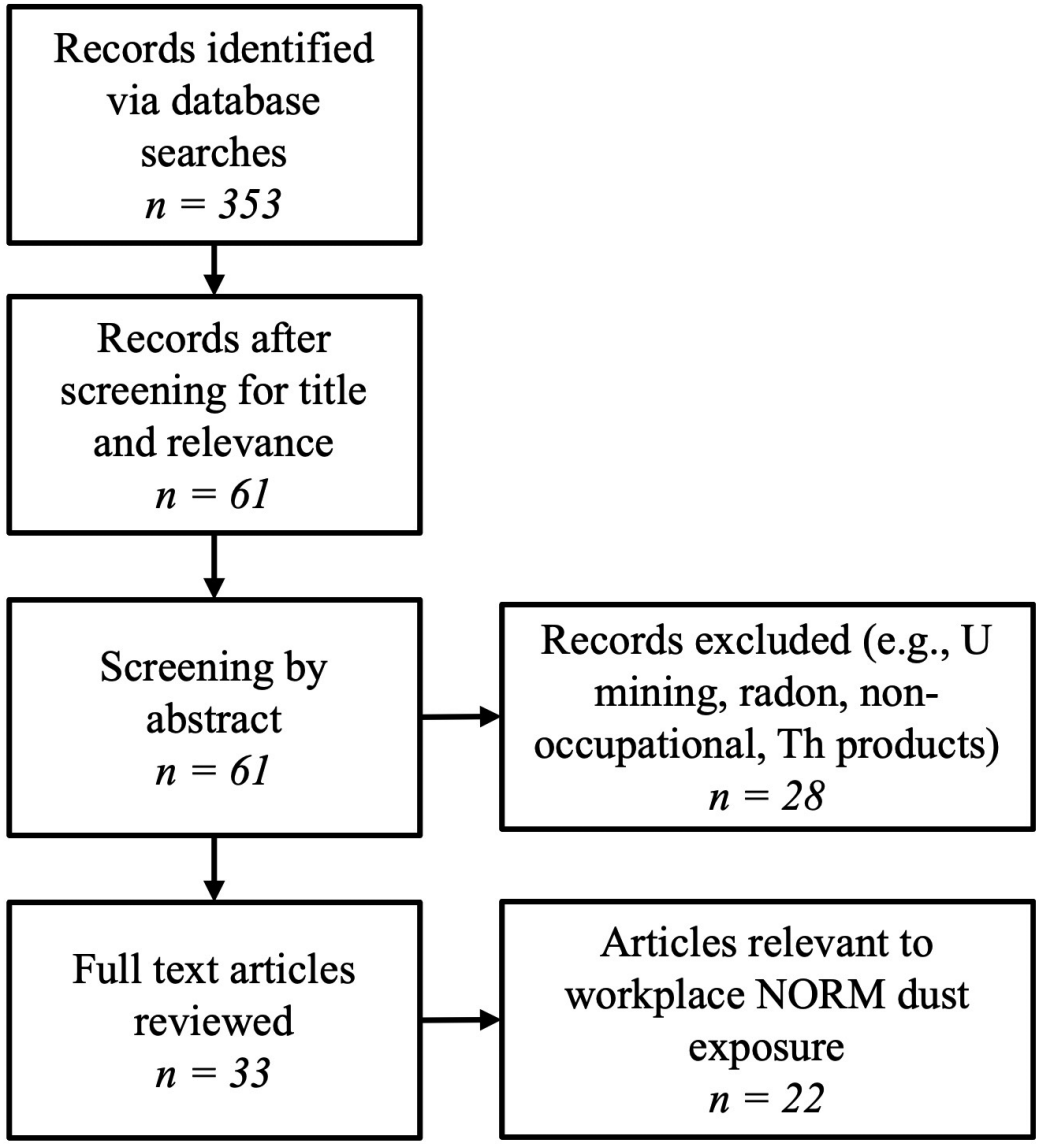
PRISMA flow diagram of NORM dust exposure review.

The secondary review involved searches of the Scopus database for studies (articles or reviews) published since 1990 using the following search term: (‘worker’) AND (‘naturally occurring radioactive material’ OR ‘monazite’ OR ‘zircon’ OR ‘ilmenite’) AND (‘air sampling’ OR ‘PAS’ OR ‘particle size’ OR ‘inhaled *activity’ OR ‘airborne dust’ OR ‘dose assessment’ OR ‘thorium exposure’). Studies that were not directly related to workplace NORM dust assessment were excluded. The method section of NORM articles flagged for review was analysed to assess how airborne radioactivity measurements were conducted, including sample heads, aerosol fractions, and use of any modifying factors. Figure 1 summarises the review process.

Supplementary searches were conducted using the IAEA International Nuclear Information System (INIS) database to identify any additional applicable journal and conference articles, and reports relating to occupational NORM dust exposure measurement. Preference was given to peer-reviewed studies; however, several non-peer-reviewed papers applicable to NORM dust have been published in the IAEA’s NORM series of conferences (https://www.iaea.org/search/google/NORM). Articles were screened primarily for relevance to NORM dust exposure applicable to mining and mineral processing industries.

Past radiation research relevant to the intake of thorium-bearing dust was reviewed, using the recent summary by Hewson et al. (2025a). A further study relating to the characterisation of inhaled dust in Western Australian mining and mineral treatment operations (Terry et al. 1998) was used to assess the relative performance of commonly used dust sample heads for airborne radioactivity.

### Dose calculation

To identify other factors that may contribute to under- or overestimation of dose, the protocols used to convert a measured radioactivity concentration to dose were reviewed. These factors are inputs to the calculations as per Equations (1) and (2):


$$\begin{array}{l} I\alpha \;\;\;=\;\;\;C\alpha \;\;\;\times \;\;\;Br\;\;\;\times \;\;\;te \end{array}$$ (1)

where *I_α_* is the total alpha activity intake into the respiratory tract (Bq_α_), *C_α_* is the average airborne alpha activity concentration in the breathing zone of a worker (Bq_α_ m^−3^), *Br* is the breathing rate of the worker, with the default set at 1.2 m^3^ h^−1^, *te* is the exposure time (h) for the period of interest.

*C_α_* is typically obtained using results from PAS undertaken in industry-defined SEGs. Conversion of the alpha activity intake (*I_α_*) to the internal radiation dose is accomplished using biokinetic and dosimetric models recommended by the ICRP (2015) according to the following:


$$\begin{array}{l} De\;\;\;=\;\;\;I\alpha \;\;\;\times \;\;\;DCFinh \end{array}$$ (2)

where *De* is the committed effective dose in mSv (referred to as the dose in this study), DCFinh is the inhalation dose conversion factor (mSv Bq_α_^−1^).

DCFinh is determined for the appropriate mix of radionuclides in the dust (eg as per thorium-to-uranium ratio), and is also dependent on particle size, specifically the activity median aerodynamic diameter—AMAD, and solubility of the inhaled dust.

Note: The AMAD is the aerodynamic diameter at which 50% of the airborne activity is associated with particles larger than the AMAD. In internal dosimetry, the AMAD is used for the simplification as a single ‘average’ value of aerodynamic diameter that is representative of the aerosol as a whole (IAEA 2018).

Analyses were conducted to evaluate the impact of different breathing rates, airborne radioactivity distribution statistical parameters (geometric vs. arithmetic mean [AM]), and dose conversion factors as a function of particle size, on the estimated dose.

## Results

### Literature review findings

The key documents providing guidance on monitoring and assessing NORM exposure are issued by various local and international radiation protection agencies, and these are summarised in Table 1.

**Table 1.** Local and international guidance for assessing internal exposure to dusts containing naturally occurring radioactive material (NORM).

Agency	Key documents identified [and content]	Reference
DEMIRS^a^	NORM-3.4 Monitoring NORM—airborne radioactivity sampling. [Provides methodology for sampling and analysis, including statistical treatment of results.]	GWA 2010
DEMIRS^a^	NORM-5 Dose assessment. [Provides methodology for assessing dose from NORM intakes.]	GWA 2023
DEMIRS^a^	Preparation of a health and hygiene management plan—guide. [Broad guidance on good OH practice.]	GWA 2018
Standards Australia	AS 3640: method for sampling and gravimetric determination of inhalable dust. [Recommends sample heads for collecting inhalable dust.]	Standards Australia 2009
ICRP^b^	Occupational intakes of radionuclides: Part 1 (ICRP Publication 130). [Comprehensive overview of monitoring and assessing the intake of radionuclides; describes the biokinetic and dosimetric models used to convert inhalation intake to dose.]	ICRP 2015
ICRP^b^	Radiological protection from naturally occurring radioactive material (NORM) in industrial processes. ICRP Publication 142. [Broad guidance]	ICRP 2019
IAEA^c^	Occupational radiation protection in the mining and processing of raw materials. RSG-1.6. [Broad guidance.]	IAEA 2004
IAEA^c^	Radiation protection and NORM residue management in the production of rare earths from thorium-containing minerals. SRS-68. [Specific to rare earths, mineral sands.]	IAEA 2011
IAEA^c^	Occupational radiation protection. GSG-7. [Recommends internal dose monitoring protocols.]	IAEA 2018

^a^Western Australian Department of Energy, Mines, Industry Regulation and Safety (www.commerce.wa.gov.au/worksafe).

^b^International Commission on Radiological Protection (www.icrp.org/).

^c^International Atomic Energy Agency (www.iaea.org/).

The review of these documents identified a lack of standardised guidance for sampling airborne radioactivity in NORM industries. The international agencies (IAEA, ICRP) provide general recommendations for the use of air sampling to determine radionuclide intake, but they do not specify sampling devices or protocols, leaving these decisions to local regulatory authorities. The IAEA, for example, provides generic advice on monitoring techniques for alpha activity in dust, with emphasis on the radiometric analysis rather than sampling techniques (IAEA 2004).

The IAEA and ICRP have contrasting views on the utility of air sampling. ICRP (2015) notes the poor correlation between PAS and bioassay samples observed in several studies and suggests that body activity measurements and/or excreta analysis are preferable for the assessment of individual intakes of airborne radionuclides and effective doses. IAEA (2018) recommends that air sampling rather than bioassay measurements is the preferred method of assessing doses for workers engaged in industrial activities involving NORM. The IAEA acknowledges that occupational hygiene samplers (eg for inhalable dust) typically underestimate the relevant aerosol fraction and suggests correction factors to be applied to minimise this bias (App. V. pp. 294–296, IAEA 2018). The correction factors were based on the work of van der Steen et al. (2004), and these authors highlight that the lung dosimetry and biokinetic models used in radiation protection (ICRP 2015) are based on the *true ambient aerosol*. The authors concluded:

1. None of the different PAS sample heads (ie those following the international occupational hygiene sampling conventions) is a perfect match for radiation protection purposes.
2. Appropriate correction factors should be applied to the inhalable or thoracic PAS results depending on the particle size dispersion characteristics (AMAD and GSD) of workplace aerosols.
3. A thoracic sample head (ie a sampler meeting the ISO 7708/EN 481 (1995) thoracic criteria) should be used for lung solubility absorption of type S (slow) and M (moderate) materials.

Van der Steen et al. (2004) recommended the following correction factors to be applied to the measured inhalable activity concentration to estimate the ambient aerosol activity concentration:

- For the default 5 µm AMAD (GSD = 2.5) aerosol, a correction factor of 1.18.
- For a 10 µm AMAD (GSD = 2.5) aerosol, a factor of 1.31.

The database searches of Scopus and INIS returned many papers relating to NORM, but relatively few directly applicable to the assessment of NORM dust exposure (Fig. 1). The Scopus search returned 353 potential articles, and this number reduced to 61 following review of title and applying exclusion criteria. Further review of abstracts and methods identified 33 potential studies with NORM dust sampling information, with 22 publications directly applicable to assessment of exposure to NORM dust. In general, the methods section in many of these studies focussed on the radiological analysis of collected samples rather than describing the sampler characteristics and aerosol fraction. Some studies didn’t measure or analyse the airborne radioactivity concentration but instead estimated intake by applying the specific activity (Bq mg^−1^) of the bulk materials being processed to the measured or an assumed gravimetric dust concentration (in mg m^−3^). Significant variability in air sampling methods was evident from the published studies as illustrated by the analysis below. Such variability complicates direct comparisons of exposure levels between studies.

Summaries of airborne dust characteristics in mineral sands separation plants in several countries have been published by the IAEA (2011); however, details of the sampling methods, and hence aerosol fractions, used to derive the mass and radioactivity concentrations for the different operations were not provided. Airborne radioactive dust studies in NORM industries, such as zircon, ilmenite, and rare earth elements (Righi et al. 2005; Haridasan et al. 2006, 2008a, 2008b; Ballesteros et al. 2008; Fathabadi et al. 2011; Zhang et al. 2021) and phosphate processing (Kim et al. 2006), typically report results from area, high-volume air samplers, not PAS, and the dust sampling instrument (and sample head) is not usually described. Other studies (Johnston 1991; Dias da Cunha et al. 2002; Lipsztein et al. 2003; Pires do Rio et al. 2008; Iwaoka et al. 2013) report results using samplers collecting a fraction of airborne dust, such as respirable dust, using cyclones or 2-stage impactors. A 37-mm open-face plastic cassette was used for dust sampling in a mineral sands plant (Hewson and Ralph 1992) and several ‘amang’ (by-product mineral from tin mining) plants (Hewson 1996), and a 7-hole sample head was used for PAS in a monazite separation plant (Terry et al. 1995) and in zircon milling plants (Hartley 2001). Positional and PAS measurements using both 7-hole and SKC plastic cyclones were used to assess NORM exposure in iron ore processing (Dal-Molin et al. 2017). An SKC ‘Button’ inhalable dust sample head was used to determine internal doses to ceramic zircon workers (Sevilla et al. 2024). A recent study collected airborne radioactive dust in monazite processing industries using PAS; however, only the sampling pump was described and not the sample head (Lee et al. 2025). In none of the above studies were sample head correction factors applied to the measured dust or airborne radioactivity concentrations.

### Review of sampler performance

The Australian Standard for inhalable dust sampling, AS 3640 (Standards Australia 2009), refers to 2 acceptable dust sample heads; namely, the 7-hole sample head and the Institute of Occupational Medicine (IOM) sample head. Both sample heads continue to be used in Australian industrial operations and in other jurisdictions worldwide. Only the 7-hole is specified for sampling long-lived alpha activity in air, and it is also listed as an acceptable sample head for many of the atmospheric contaminants encountered in mining (ie for metals) (GWA 2024).

**Figure 2.**
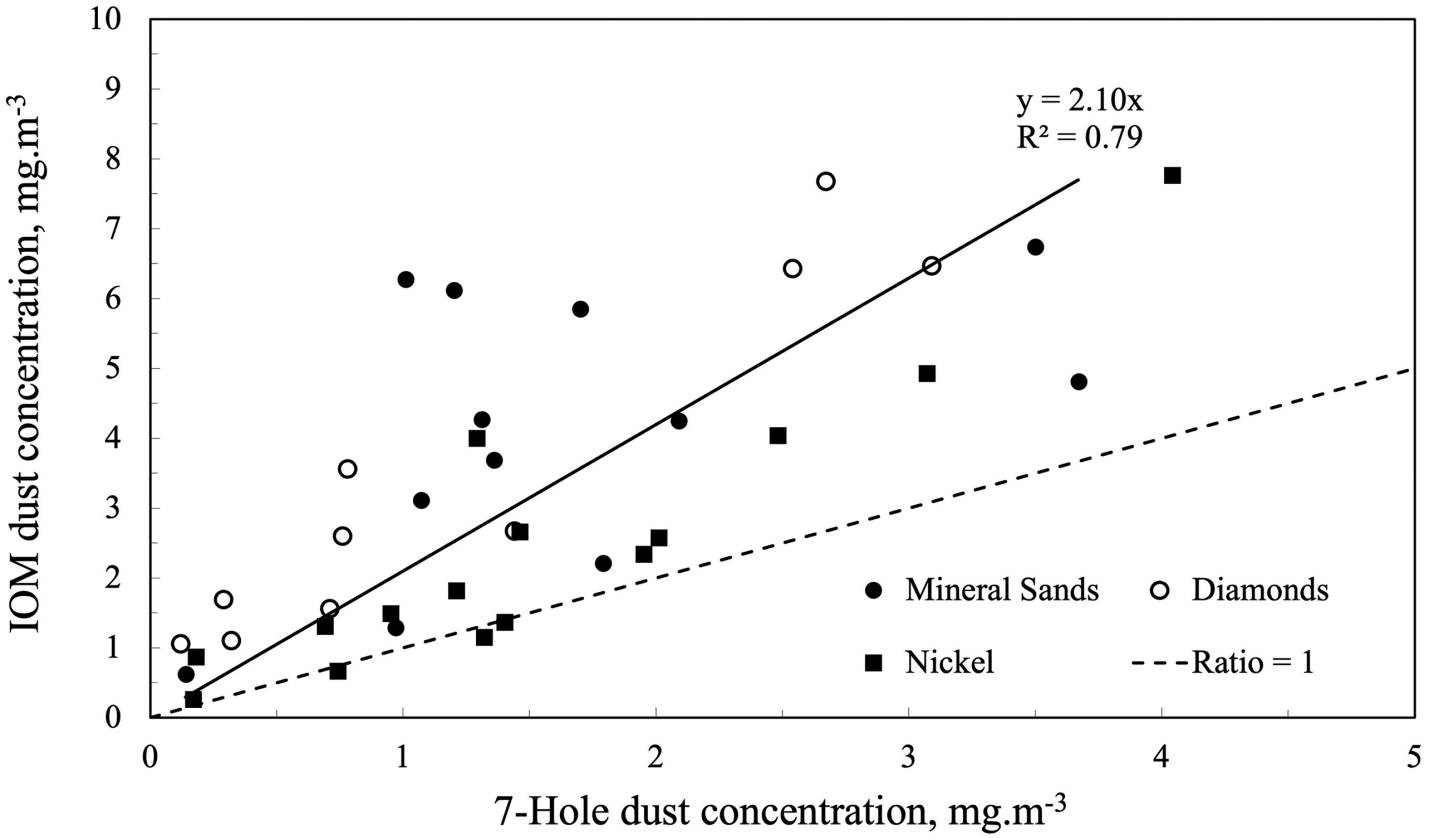
IOM sampler vs 7-hole sampler comparison at three different Western Australian mineral mining or processing sites (adapted from Terry et al. 1998). Trend line and regression statistics relate to data obtained from mineral sands workers.

The potential for the 7-hole to underestimate the inhalable dust fraction, especially when large particles are present, was identified during comparative studies with the IOM sample head in the Western Australian mining industry in the 1990s (Terry et al. 1998). Figure 2, interpreted from data presented in Terry et al. (1998), summarises the results of the side-by-side comparison (*n* = 37) of the 7-hole and IOM sample heads at three Western Australian mineral sectors: mineral sands, nickel, and diamond mining. The IOM sampler recorded higher dust concentrations than the 7-hole in 95% of the tests, and a least-squares best fit (IOM: 7-hole) from the comparative tests (*n* = 12) on mineral sands dry plant workers was 2.1 (*r*^2^ = 0.79). All 3 mineral sectors were characterised by workplace dust atmospheres with mass median aerodynamic diameters (MMADs) >15 µm as measured by personal cascade impactors. Additionally, the study highlighted the potential for further underestimation in the case of airborne radioactivity sampling due to wall losses. Alpha particle counting is typically restricted to the dust collected on the filter, and no accounting is made for airborne dust that had adhered to the sampler walls. This wall loss issue applied to both the IOM and 7-hole sample heads.

Borsh et al. (2019) report that the IOM is considered the ‘gold standard’ for sampling the inhalable fraction, and the UK Health and Safety Executive (2014) recommends the IOM as the preferred method of sampling the inhalable aerosol. The significant difference in collected dust between the IOM and 7-hole may be attributed to the IOM’s higher collection efficiency for larger particles, resulting in greater dust capture (Kenny et al. 1997). The IOM has a single 15-mm orifice while the 7-hole has seven equally spaced 4-mm orifices. Kenny et al. (1997) tested the 7-hole in wind tunnel experiments and found that the 7-hole significantly under-sampled the inhalable fraction, particularly at aerodynamic diameters >10 µm. The results shown in Fig. 2 support the findings of Kenny et al. (1997) and those of Vincent (1999), who also found a tendency for greater bias (between the different sample heads) for workplaces where the aerosol is expected to be coarser. Vincent reported an average IOM ratio of 2.2 to various legacy ‘total’ dust sample heads from 915 comparisons across several countries, industries, and workplace aerosols.

The comparative sample head studies (Fig. 2) in Western Australia were performed >25 yr ago. A recent literature review (Hanlon et al. 2021) identified only 1 other recent workplace study comparing the IOM and 7-hole, which was conducted in a European rubber manufacturing facility (de Vocht et al. 2006). This study found that the IOM provided a good match to the inhalable fraction, but the 7-hole under-sampled by a factor of 1.5. The higher factor in the mineral sands study (Terry et al. 1997) is likely due to mineral dust being coarser than the dust and fumes encountered in rubber manufacturing.

### Analysis of dose calculation parameters

The recommended default parameter values for inhalation of NORM dust are 5 µm AMAD, solubility ‘Type S’ (slow absorption) (IAEA 2018) and a breathing rate of 1.2 m^3^ h^−1^, based on ‘light’ activity (ie 31% rest at 0.54 m^3^ h^−1^ and 69% light exercise at 1.5 m^3^ h^−1^) (ICRP 2015). The dust solubility classification determines the clearance half-time (absorption to the blood) of dust particles deposited in the lung. Mineral sands dusts, together with many other dusts arising from the mining and treatment of NORM materials, are considered relatively insoluble and are assigned to Type S (IAEA 2018). No adjustment to the solubility classification was considered justified in this study.

#### Breathing rate (*Br*)

The default rate may underestimate intake for workers performing physically demanding tasks or work in hot environments. Increasing the breathing rate to 1.5 m³ h⁻¹, which reflects moderate physical exertion, results in a 25% increase in estimated intake. This adjustment accounts for the higher ventilation rates associated with climbing stairs, handling equipment, and other tasks common in mining and mineral processing environments. The ICRP (2003, pp. 100–101) indicates an even higher average breathing rate over the work shift of 1.7 m3 h⁻¹ for males conducting heavy work such as construction workers.

#### Particle size (AMAD)

The ICRP’s recommended default AMAD of 5 µm for occupational exposure is largely based on the review by Dorrian and Bailey (1995) and is not representative of the particle size distributions observed in Western Australian mineral processing plants. Particle size measurements using a personal cascade impactor worn by workers at some Western Australian surface mining and mineral processing plants are summarised in Table 2. These mineral sectors were characterised by an AMAD or MMAD of the inhaled dust of typically 15–20 µm. In mineral sands operations, the AMAD tends to be slightly smaller than the MMAD because monazite (the most radioactive mineral) tends to be softer and finer than the other mineral sand constituents (eg ilmenite and zircon) (Hewson 1997). The consistency of the median particle sizes experienced by Western Australian mineral sands workers across the four studies listed in Table 2 is due to the similar types and layouts of separation plant equipment in multi-story buildings and the use of similar operational work practices. No further studies on particle size distribution measurements in the Western Australian minerals industry have been publicly reported since Terry et al. (1998).

**Table 2.** Particle size measurements on workers in Western Australian mineral treatment plants and surface mining operations.^a^

Mineral sector	Work Activity	Test year	No. tests	MMAD^b^ (AMAD), µm	GSD^c^
Mineral sands^d^	Dry plant operators	1993	49	(15.7)	2.9
Mineral sands^e^	Dry plant operators	1995	25	18.0	2.1
Mineral sands^f^	Monazite plant operators	1995	5	(14.0)	3.0
Mineral sands^g^	Dry plant operators	1997	3	17.7	2.3
Nickel^g^	Surface mine workers	1997	8	18.6	2.2
Industrial minerals^g^	Dry plant operators	1997	12	21.3	2.0
Bauxite/ alumina^g^	Refinery operators	1997	8	22.5	1.6
Iron ore^g^	Surface mine workers	1997	27	16.3	2.3

^a^Marple 298 personal cascade impactor was used to determine median particle size in all tests listed.

^b^MMAD—Mass median aerodynamic diameter. AMAD = activity median aerodynamic diameter. AMADs determined via alpha particle counting of impactor plates.

^c^GSD—Geometric standard deviation.

^d^Data from Koperski (1993); reported as AMAD.

^e^The raw data from 25 tests were directly provided to one of the authors by the company.

^f^Data from measurements on five consecutive days; reported as AMAD (Terry et al. 1995).

^g^Data extracted from a mining industry research study (Terry et al. 1998).

Applying a workplace-specific AMAD of 10 µm, instead of the default 5 µm, reduces DCFinh from 0.0148 to 0.0080 mSv Bq_α_^−1^ for Type S, NORM dust with a thorium-to-uranium ratio of 10:1 (GWA 2023). This 1.85-fold reduction reflects the increase in rapid clearance of large particles from the nose and upper airways and the reduced penetration of particles into the alveolar region, resulting in less dose per intake.

#### Arithmetic versus geometric mean

Current local regulatory guidelines (GWA 2010, 2023) use the geometric mean (GM) airborne radioactivity concentration for the applicable SEG(s) to calculate worker intake. However, cumulative exposure to alpha-emitting radionuclides is better represented by the AM, which reflects long-term health risk. For a well-defined SEG with a lognormal distribution of radioactivity concentrations and a geometric standard deviation (GSD) of 2, the AM is 1.27 times higher than the GM, indicating that using the GM may underestimate cumulative intake by 27%. The extent of underestimation increases to 52% if the SEG has a GSD of 2.5.

The literature review revealed inconsistency with the reporting of airborne radioactivity concentrations in NORM operations, with both normal and lognormal distribution parameters being quoted, with a potential commensurate impact on doses.

## Discussion

Accurate assessment of radiation doses from the inhalation of NORM dust is essential for protecting worker health. The literature review revealed considerable variability in the way NORM dust exposures are measured across industry sectors and regions; particularly, the lack of detail around the aerosol fraction collected.

### Sampler considerations and treatment of wall losses

Sample head comparison studies (Kenny et al. 1997; Hanlon et al. 2021) have highlighted that sample head efficiency (or bias) curves as a function of particle size (aerodynamic diameter) and ambient air velocity can translate to significantly different masses of collected dust depending on the sample head used. In the case of sampling for radioactive aerosols, this translates to different activity concentrations being recorded and hence has a direct impact on the dose assessment (as per equations (1) and (2)). The 7-hole was originally recommended for sampling inhaled radioactive dust owing to a concern that the IOM captures significant dust on the inside walls of the sampling cassette (GWA 2010), which was considered problematic for subsequent analysis of radioactivity by alpha particle counting.

The current Australian Standard for inhalable dust sampling, AS 3640 (Standards Australia 2009), has been flagged for review, which should remove the recommendation for the use of the legacy 7-hole sample head and include updated guidance on appropriate inhalable dust samplers. Such a revision would require a consequential change to regulatory guidelines (GWA 2010, 2024) for sampling of airborne radioactivity.

A potentially significant issue is the lack of accounting for sampler wall losses in the analysis of radioactivity of the dust captured by the sample head. White et al. (2024) also emphasises this issue and the potential to significantly underestimate the true inhalable dust concentration (and hence, dose). A more accurate assessment would require “washing” the wall-deposited material and redepositing it onto a second filter paper for subsequent analysis, thereby increasing the sampling analysis effort.

Notwithstanding the identified issues associated with the sampling bias of the 7-hole, its long-term (>30 yr) use for PAS in Western Australia NORM operations has promoted consistency in exposure measurements across different sites. However, any future health effect studies of NORM workers should be based on revised assessments of historical intakes using appropriate sampler adjustment factors.

### Sampler adjustments for radioactive aerosols

A further consideration relates to potential correction factors, as recommended by the ICRP (2002) and IAEA (2011, 2018), that should be made to industry PAS measurements obtained from inhalable dust sample heads to reflect what is relevant from a radiation protection and dosimetry perspective. The use of correction factors for airborne radioactivity concentrations does not yet seem to be a routine practice given the findings from the literature review. In the Western Australian mineral sands industry, characterised by a 10 µm AMAD aerosol (Table 2), the implication is that a total adjustment factor of 2.75 should be applied to activity concentrations currently recorded by a 7-hole sample head (ie a factor of 2.1 to convert 7-hole concentration to obtain the inhalable concentration (as shown in Figure 2) and a further factor of 1.31 to obtain the ambient aerosol concentration as per ICRP/ IAEA guidance). Adjustment factors would also apply to other NORM industries if similar PAS protocols were used to assess airborne activity intake.

In the future, thoracic dust sampling may be most appropriate for operations involving NORM associated with low-solubility mineral matrices. Witshger (2005) found that thoracic sample heads had the least bias (ie lowest under- or overestimation of dose) compared to other samplers when applying the DCFinh for a 5 µm AMAD to the collected dust fraction. Use of a thoracic sample head should also assist in reducing wall losses as observed in inhalable sample heads when used in coarse dust atmospheres.

It is noteworthy that following exposure to coarse NORM aerosols, most of the inhaled activity is deposited in the nasal passages and upper airways. These deposits are rapidly cleared either externally to the environment (via the nose or mouth) or by swallowing and subsequent faecal excretion and hence have little radiological significance. For insoluble NORM dust (and other insoluble mineral dusts), the important aerosol fraction is likely to be the thoracic fraction.

### Particle size and breathing rates

The use of default assumptions regarding breathing rate (1.2 m³ h⁻¹) and particle size (5 µm AMAD), and the choice of statistical mean concentration (AM vs. GM) may contribute to under- or overestimation of dose. It is important to use workplace-specific particle size distributions when assessing dose from intake, given the dependence of DCFinh on AMAD. The use of a default 5 µm AMAD is overly conservative for NORM dust exposures in mineral sands operations (as per Table 2), and it may also be for other industries where NORM-containing minerals are not subject to crushing and grinding processes. The particle size distribution is also a key factor in the collection characteristics of the dust sample head used.

In relation to breathing rates, no evidence of the use of alternate breathing rates in exposure assessment for NORM workers has been found in the literature. Representative rates should be established for each SEG to further improve the accuracy of intake estimates.

### Combined impact of parameter adjustments:

Table 3 summarises the combined impact of using an alternative sample head better matching the inhalable aerosol convention, applying ICRP/ IAEA recommended correction factors for the measured AMAD, increasing the breathing rate, and using the AM for SEG exposures. The overall impact of applying the adjustment factors is a 2.3-fold increase in estimated dose compared to current protocols. This implies current doses derived from PAS are not accurate and understate the radiological risk. The uncertainty on the total impact is estimated as GSD = 2.5, derived by combining the GSDs associated with each parameter listed in Table 3. The largest contributions to dose underestimation arise from the change in sample head type (2.1), applying the recommended sampler efficiency correction factor (1.31), and the use of AMs (1.27). The reduction in DCFinh due to the larger particle size (10 µm AMAD vs. 5 µm AMAD) partially offsets these increases.

**Table 3.** Potential underestimation of radioactivity intake due to current sampling and exposure assessment protocols used in Western Australian mining operations.

Method/ Parameter	Default	Alternative	Factor^a^	Uncertainty (GSD)
Dust sampling method	7-hole sample head	IOM sample head^b^	2.1	1.75
Airborne activity concentration adjustmentc	1	1.31	1.31	-^d^
Breathing rate	1.2 m^3^ h^-1^	1.5 m^3^ h^-1^	1.25	1.2
SEG mean^e^	Geometric	Arithmetic	1.27	1.2
AMAD (µm)^f^	5	10	0.54	2.0
Total impact			2.3	2.5^g^

^a^Refer text for discussion and derivation of each factor.

^b^Or other sampler with sampling efficiency closely matching the ISO inhalable dust convention.

^c^Specifically for workplaces involving exposure to naturally occurring radionuclides, a correction factor is recommended for different samplers to minimise the potential for samplers to underestimate the inhaled activity (V.39, pp. 295–296, IAEA 2018). The factor of 1.3 is for an inhalable sampler used to sample aerosols with an AMAD of 10 µm.

^d^This factor is dependent on the particle size distribution (AMAD and GSD) of the aerosol, and the sampling efficiency of the PAS sample head.

^e^Assuming SEG distribution GSD of 2 and using AM = GM × exp(½ (lnGSD)2).

^f^Based on DCFinh (in mSv.Bq_ɑ_^−^^1^) for a NORM dust with a Th:U weight ratio of 10:1 (GWA 2023).

^g^The total uncertainty is given by: GSDtotal = exp(Vtotal)½, where Vtotal = Σ(ln GSDi)2. This assumes independent parameters and the uncertainties being lognormally distributed.

The total impact will be greater if the aerosol AMAD is less than 10 µm, the average breathing rates are higher than assumed (eg if workers are involved in strenuous tasks), or SEG concentration distributions have GSDs >2. The impact will be less if the NORM dust is more soluble than assumed, because the assumption of an insoluble mineral matrix is more conservative from a lung dosimetry perspective (ie longer retention of dust in the lungs resulting in protracted alpha particle irradiation).

### Findings from bioassay studies

A summary of past research in the Western Australian mineral sands industry highlighted that industry reported PAS-derived intakes were two to three times lower than intakes derived from various in vivo and in vitro bioassay measurements. (Hewson et al. 2025a). A recent re-analysis of historical urine bioassay data using the latest biokinetic and dosimetric models showed an average ratio of bioassay intake to PAS intake of 2.5 for the mineral sands workers tested (Hewson et al. 2025b). The authors concluded that a critical review of the appropriateness of PAS and analysis strategies was needed. The results from the bioassay studies suggest that the adjustments proposed in this paper should provide a more accurate estimate of worker exposure.

The bioassay studies were conducted over 30 yr ago, and further studies should be considered, if feasible, to confirm the adequacy of contemporary PAS strategies and to assess individual variability in work and hygiene habits.

### Broader implications for NORM and industrial dust exposure

While this study focuses on impacts to exposures in the Western Australian minerals industry, the findings have broader implications for other NORM industries where workers are exposed to coarse aerosols. The choice of inhalable dust sample head is likely to affect exposure assessments in these industries, particularly when dust particle sizes exceed 15–20 µm. Additionally, the study emphasises the importance of accounting for sampler wall losses and understanding sampler efficiency as a function of workplace particle size distributions. Future research should investigate the performance of alternative samplers, such as thoracic samplers, which may provide more accurate measurements of the radiologically significant dust fraction following exposure to NORM associated with low-solubility mineral dusts.

## Conclusions

This study identified critical shortcomings in air sampling methods and dose assessment protocols used to estimate worker exposure to NORM dust in the Western Australian minerals industry. The 7-hole sample head, which is currently recommended for PAS, underestimates the true airborne radioactivity concentrations by up to two-fold (cf IOM sampler) due to its reduced efficiency in capturing larger dust particles. Additional factors, including the use of a default particle size of 5 µm AMAD, a breathing rate of 1.2 m³ h⁻¹, and reliance on GMs for SEG assessments, further contribute to dose under- or overestimation. The combined effect of these factors results in a 2.3-fold underestimation of internal radiation doses using current protocols.

To improve the accuracy of dose assessments, this study recommends the following measures:

1. **Replace the 7-hole sample head** with a more efficient inhalable dust sampler (eg a sampler more closely matching the ISO inhalability convention) or a thoracic sampler, to better capture the radiologically relevant aerosol particles encountered in mineral processing environments.
2. **Adjust dose calculation parameters** by using a site-specific AMAD (eg 10 µm for mineral sands processing), increasing the breathing rate to 1.5 m³·h⁻¹ or higher depending on worker activity levels, and applying AMs for SEG assessments to provide a more accurate estimate of cumulative exposure.
3. **Investigate sample head wall losses** and develop methods to account for alpha activity retained on sample head walls, which may further contribute to underestimation of airborne radioactivity concentrations.

These recommendations are essential for ensuring that worker exposure to NORM dust is accurately quantified, supporting effective radiation protection measures and aligning industry practices with current scientific evidence. Studies reporting NORM-related dust exposures should specify the aerosol sampling equipment and analysis methodology to enable meaningful exposure comparisons across NORM industries. The findings also have broader implications for other industries where workers are exposed to coarse aerosols. Future research should focus on validating these recommendations through workplace studies and bioassay monitoring, where feasible, to confirm their effectiveness in improving exposure assessments and protecting worker health.

## Data Availability

The data underlying this article are available in the article and from the publicly available reports and articles cited in the article.
